# Loss of cholinergic input to the entorhinal cortex is an early indicator of cognitive impairment in natural aging of humans and mice

**DOI:** 10.21203/rs.3.rs-3851086/v2

**Published:** 2024-02-29

**Authors:** Mala R. Ananth, John D. Gardus, Chuan Huang, Nikhil Palekar, Mark Slifstein, Laszlo Zaborszky, Ramin V. Parsey, David A. Talmage, Christine DeLorenzo, Lorna W. Role

**Affiliations:** National Institutes of Health; Stony Brook Medicine; Emory University School of Medicine; Stony Brook Medicine; Stony Brook Medicine; Rutgers University; Stony Brook Medicine; National Institutes of Health; Stony Brook Medicine; National Institutes of Health

**Keywords:** Cholinergic, Entorhinal Cortex, Aging, Cognition, Translational, PET, MRI

## Abstract

In a series of translational experiments using fully quantitative positron emission tomography (PET) imaging with a new tracer specific for the vesicular acetylcholine transporter ([^18^F]VAT) in vivo in humans, and genetically targeted cholinergic markers in mice, we evaluated whether changes to the cholinergic system were an early feature of age-related cognitive decline. We found that deficits in cholinergic innervation of the entorhinal cortex (EC) and decline in performance on behavioral tasks engaging the EC are, strikingly, early features of the aging process. In human studies, we recruited older adult volunteers that were physically healthy and without prior clinical diagnosis of cognitive impairment. Using [^18^F]VAT PET imaging, we demonstrate that there is measurable loss of cholinergic inputs to the EC that can serve as an early signature of decline in EC cognitive performance. These deficits are specific to the cholinergic circuit between the medial septum and vertical limb of the diagonal band (MS/vDB; CH1/2) to the EC. Using diffusion imaging, we further demonstrate impaired structural connectivity in the tracts between the MS/vDB and EC in older adults with mild cognitive impairment. Experiments in mouse, designed to parallel and extend upon the human studies, used high resolution imaging to evaluate cholinergic terminal density and immediate early gene (IEG) activity of EC neurons in healthy aging mice and in mice with genetic susceptibility to accelerated accumulation amyloid beta plaques and hyperphosphorylated mouse tau. Across species and aging conditions, we find that the integrity of cholinergic projections to the EC directly correlates with the extent of EC activation and with performance on EC-related object recognition memory tasks. Silencing EC-projecting cholinergic neurons in young, healthy mice during the object-location memory task impairs object recognition performance, mimicking aging. Taken together we identify a role for acetylcholine in normal EC function and establish loss of cholinergic input to the EC as an early, conserved feature of age-related cognitive decline in both humans and rodents.

## I. Introduction

Acetylcholine is a key neuromodulator in the brain that is critical for attention, wakefulness, mood, and memory^1^. Cholinergic neurons (neurons that synthesize and release acetylcholine), coordinate neuronal activity brain-wide to promote attention to salient stimuli and facilitate learning^2^. Basal forebrain cholinergic neurons (BFCNs) span the entire rostro-caudal forebrain and send wide-spread projections to much of the brain^3^. The broad reach of these projections coupled with their functional organization grant cholinergic neurons highly flexible, context-specific control over cortical dynamics, making a strong case for the functional importance of acetylcholine in cognitive behaviors^4^.

Post-mortem analyses reveal loss of BFCNs and fragmentation of cholinergic projections in pathological aging conditions such as Alzheimer’s disease (AD)^5–7^. What these studies lack is an understanding of when changes to the cholinergic system occur and the importance of these changes to changes in cognition. Addressing these questions requires an early assessment of the integrity of the cholinergic system.

One of the brain regions affected earliest by age is the entorhinal cortex (EC)^8^. The EC serves as the primary input and output structure from the hippocampal formation and thus is essential for memory^9,10^. Histopathological studies reveal that accumulation of tau pathology associated with AD begins in the EC^11,12^. In addition, structural and functional alterations to the EC precede and are predictive of future cognitive impairment^13–16^. Functional deficits in the entorhinal cortex, resulting in deficits in object location memory, and processing of complex objects are among the earliest reported in AD progression^17–19^. The EC receives cholinergic input from a cluster of anteriorly positioned BFCNs^1,3,20^. Whether a compromised cholinergic system influences early cognitive changes associated with EC function and integrity is not clear.

In this study we ask whether there is an association between the status of cholinergic input to the EC and EC cognitive ability in healthy, elderly volunteers. Next, in healthy aging mice, we evaluate the integrity of cholinergic inputs to the EC, and involvement of this circuit in EC-related object location memory. Finally, we compare these findings to a mouse model with accelerated amyloid and tau pathology. Our findings demonstrate that in both humans and mice, compromised cholinergic input to the EC occurs early in the aging process, and is predictive of decreased cognitive performance, in particular, on tasks that require the EC. Genetic susceptibility to early Alzheimer’s disease accelerates the time-course of impaired EC cholinergic integrity. We find deterioration of EC cholinergic circuits is an early and shared mechanism in natural and pathological aging.

## II. Results

To gain better insight into the relationship between integrity of the cholinergic system and cognitive decline we asked: 1) whether alterations to the basal forebrain cholinergic system are an early feature in the aging process in the context of EC-related functions and 2) what is the association between an intact cholinergic system and intact cognition.

### Entorhinal cortex related functions are diminished in older adults with cognitive impairment.

**Figure 1:**
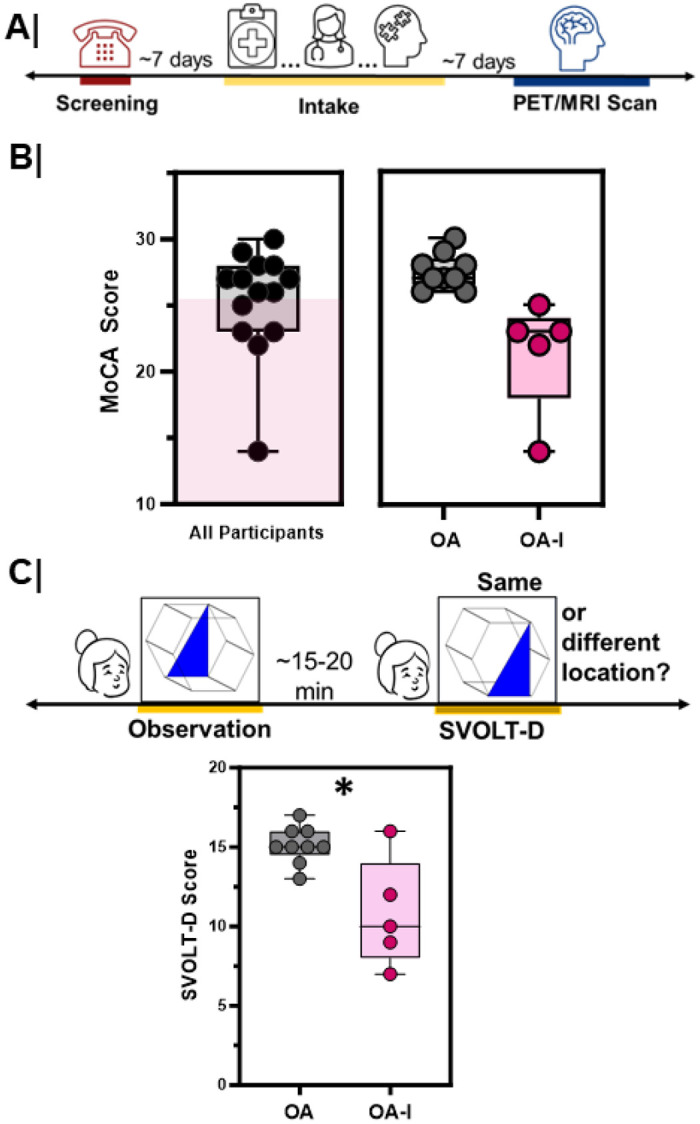
Delayed object location memory scores are lower in older adults with cognitive impairment **A**. Schematic workflow of human studies. Volunteers underwent a phone screen for initial exclusion criteria. Participants that met criteria were invited for the intake session. Each participant underwent a clinical interview, physical examination, and cognitive testing. After the intake session, participants underwent a simultaneous PET/MRI scan session. **B**. (left) Montreal Cognitive Assessment (MoCA) score across all participants was divided using the clinical threshold of 25 (top of magenta shaded box) resulting in two groups for analysis: older adults with intact cognition (**OA, Grey**, n=9) and older adults with cognitive impairment (**OA-I,Magenta**, n=5). **C**. (top) Experimental workflow highlighting the object location learning task within the Penn computerized neurocognitive battery that is administered during the intake session; (bottom) Delayed-Spatial Visual Object Learning Task (SVOLT-Delayed) between OA and OA-I participants (*, p=0.01). Older adults = **Grey**; Impaired, older adults = **Magenta**.

To answer questions about the relationship between cholinergic system integrity and cognition, we recruited older adult volunteers (Table 1) who were active in the community, physically healthy, devoid of co-morbid mental health concerns, and had no contraindications to PET and MRI scanning procedures. None of these volunteers had clinical diagnosis of cognitive impairment (Figure 1A).

During the intake session we evaluated the cognitive status of each participant using the Montreal Cognitive Assessment (MoCA, Figure 1B left), a sensitive clinical assessment for mild impairments in cognition^21^. Based on their intake session MoCA score we divided our participants into two groups, older adults (OA) and impaired, older adults (OA-I) (Figure 1B, right). All participants also underwent a standardized neurocognitive battery, the Penn Computerized Neurocognitive Battery (PennCNB), designed to assess cognitive status across multiple functional domains^22^.

The first question we asked was whether EC-related cognitive functions were altered in older adults with minor cognitive impairments. Given the importance of the EC to object location memory^19,23,24^, we used the spatial visual object learning task (SVOLT and SVOLT-delayed) within the PennCNB battery to assess EC function in our participants. SVOLT tasks test memory for complex figures. During the SVOLT observation period, participants are presented with and asked to remember a series of three-dimensional shapes with shaded sub-regions (Figure 1C, top). Immediately after the observation period, during the SVOLT test, objects from the learning set were intermingled with new objects with different shaded regions. For each presented object, participants were asked to recall whether the object was the same or different than what was presented during the observation session. For SVOLT-delayed task (SVOLT-D) the observation and recall sessions were separated by a 15–20-minute delay during which time participants performed other cognitive tasks. Participants were re-presented with a series of objects, some new and some old, in the SVOLT-D. We quantified the number of correct responses during the immediate and delayed testing session for participants in each group. OA-I participants performed significantly worse on the SVOLT-D task than their age-matched, OA counterparts (Figure 1C, bottom, OA vs. OA-I, p=0.01). In contrast, performance did not significantly differ on the immediate SVOLT test (Figure S1A; OA vs. OA-I, p=0.80). When we assessed the change in performance from immediate to delayed SVOLT session, we found that OA-I participants had a greater change in SVOLT performance score than OA individuals (Figure S1B, OA vs. OA-I, p=0.005).

### Entorhinal cortex cholinergic density is lower in older adults with cognitive impairment and correlated with spatial memory performance.

**Figure 2:**
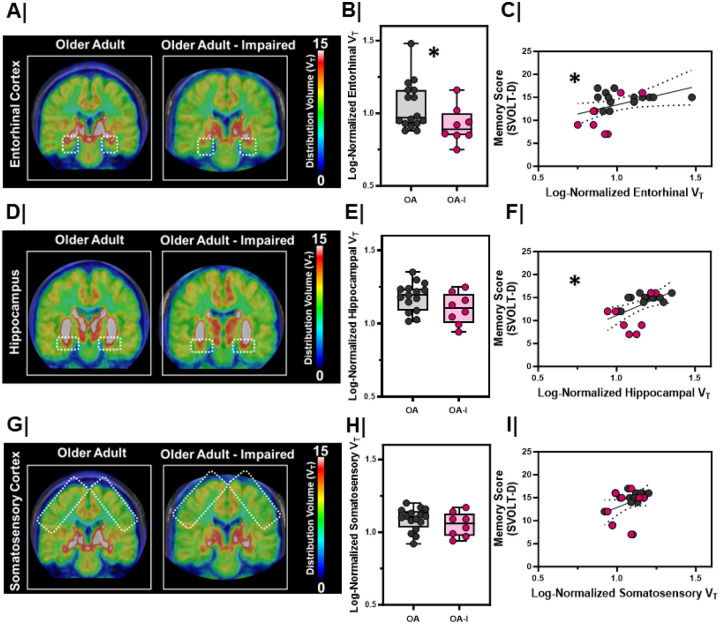
Entorhinal cortex VAT density is lower in older adults with cognitive impairment A/D/G. Averaged maps of VAChTdensity at each voxel from (left) older adults (n=8) vs. (right) impaired, older adults (n=4) at the level of the **A.** entorhinal cortex, **D.** hippocampal formation, **G.** somatosensory cortex. Color bar represents distribution volume (VT). **B/E/H.** Log-normalized right and left hemisphere VTbetween older adults (OA, n=8) and impaired, older adults (OA-I, n=4) in the **B.** entorhinal cortex (*, p=0.04) , **E.** hippocampal formation (p=0.10), **H.** somatosensory cortex (p=0.34). Older adults = **Grey**; Impaired, older adults = **Magenta.** Hemispheres plotted individually. **C/F/I.** Correlation with linear regression (black line) comparing the relationship between [18F]VAT distribution volume and delayed memory score (SVOLT-D) in the **C.** entorhinal cortex (*, rs=0.47, p=0.03, linear regression significantly non-zero; confidence interval marked by dashed black lines), F. hippocampal formation (*, rs=0.54, p=0.008, linear regression significantly non-zero; confidence interval marked by dashed black lines), **I.** somatosensory cortex (NS, rs=0.13, p=0.09, linear regression not significant; confidence interval marked by dashed black lines). Older adults = **Grey**; Impaired, older adults = **Magenta.** Hemispheres plotted individually.

Next, we asked whether OA-I participants displayed differences in the synaptic integrity of cholinergic inputs in the EC. We quantified the density of the vesicular acetylcholine transporter (VAChT) *in vivo* using [^18^F]VAT positron emission tomography (PET) (Figure 2). [^18^F]VAT specifically and selectively binds to VAChT, allowing us to quantify the integrity of the cholinergic system *in vivo*^25–27^. Linear mixed models were fit with [^18^F]VAT regional distribution volume (proportional to density) as the model outcome with group (OA or OA-I), sex (M or F), and hemisphere (R or L) as fixed effects. We found a significant main effect of group (with no main effect of sex or hemisphere), where EC distribution volumes were lower in OA-I participants compared to OA participants (Figure 2A/B, p=0.04). In contrast, distribution volumes in other areas (fit in individual models) such as the hippocampus (Figure 2D/E) or in the somatosensory cortex (Figure 2G/H) did not differ between groups (hippocampus, p = 0.10; somatosensory cortex, p = 0.34).

To directly compare the relationship between cholinergic system integrity and SVOLT performance, we next fit a model with SVOLT-D performance as the outcome and EC distribution volume as a fixed effect. Larger EC distribution volume positively correlated with higher performance on the SVOLT-D task (Figure 2C, r_S_ = 0.47, p = 0.03). We also found that hippocampal distribution volume positively correlated with SVOLT-D performance (Figure 2F, r_S_ = 0.54, p = 0.008). In contrast, somatosensory cortex distribution volume was not significantly related to performance on the SVOLT task (Figure 2I, r_S_ = 0.13, p = 0.09).

### MS/vDB (CH1/2) VAChT density is lower in older adults with cognitive impairment.

**Figure 3:**
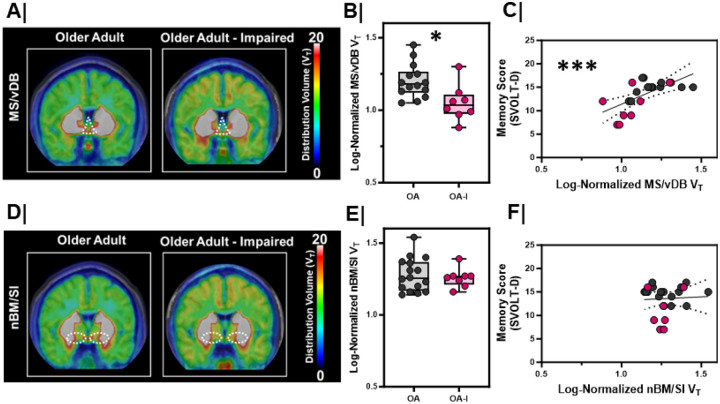
MS/vDBVAT density is lower in older adults with cognitive impairment **A/D.** Averaged maps of VAChTdensity at each voxel from (left) older adults (n=8) vs. (right) impaired, older adults (n=8) at the level of the **A.** medial septum/diagonal band (MS/vDB; CH1/CH2), **D.** nucleus basalis/substantia innominata(NBM/SI, CH4p). Color bar represents distribution volume (VT). **B/E.** Log- normalized right and left hemisphere VT between older adults (OA, n=8) and impaired, older adults (OA-I, n=4) in the **B.** MS/vDB (*, p=0.01), **E.** NBM/SI (NS, p=0.34). Older adults = **Grey;** Impaired, older adults = **Magenta**. Hemispheres plotted individually. **C/F.** Correlation with linear regression (black line) comparing the relationship between [18F]VAT distribution volume and delayed memory score (SVOLT-D) in the C. MS/vDB (***, rs=0.42, p<0.001, linear regression significantly non-zero; confidence interval marked by dashed black lines),**F.** NBM/SI (NS, rs=0.03, p=0.80, linear regression not significant; confidence interval marked by dashed black lines). Older adults = **Grey**; Impaired, older adults = **Magenta.** Hemispheres plotted individually.

In rodents and non-human primates, cholinergic neurons in MS/vDB region innervate the EC, the hippocampal subfields and the prefrontal cortex^3,28,29^. Using a previously validated BFCN subregion atlas^30^, we evaluated MS/vDB (CH1/2), hDB (CH3), and nBM/SI (CH4p) distribution volumes across our groups (Figure 3/S2). Linear mixed models were fit with MS/vDB distribution volume as the model outcome with group (OA or OA-I), sex (M or F), and hemisphere (R or L) as fixed effects. We found that MS/vDB distribution volume was significantly lower in OA-I participants compared to OA participants (Figure 3A/B, p=0.01) with no effect of sex or hemisphere. This group difference was not observed in individually fit model group comparisons for nBM/SI (Figure 3D/E, p=0.34) or hDB (Figure S2A, p=0.16) distribution volumes.

To directly compare the relationship between cholinergic integrity in BFCNs and SVOLT performance, we fit a model with SVOLT-D performance as the outcome and MS/vDB distribution volume as a fixed effect. We found larger MS/vDB distribution volumes corresponded with higher performance on the SVOLT-D task (Figure 3C, r_S_ = 0.67, p<0.001). In contrast, there was no relationship between SVOLT-D performance and distribution volume in the nBM (Figure 3F, rS = 0.05, p = 0.82) or hDB (Figure S2B, r_S_ = 0.16, p = 0.47).

### The circuit between the MS/vDB and EC is structurally impaired in older adults with cognitive impairment.

**Figure 4:**
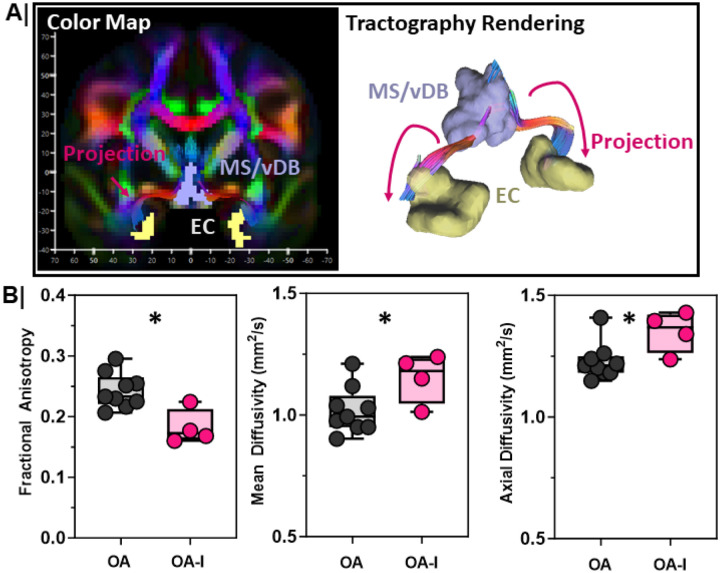
MS/vDBto entorhinal cortex connectivity is impaired in older adults with cognitive impairment **A. (Left)** Representative color map from an OA participant in coronal plane where fiber direction is represented by color. (**Right**) Corresponding 3D tractography depicts the reconstructed tract (magenta) between the MS/vDB (lavender) and EC (yellow) regions within which diffusion metrics were measured between older adults (OA, n=9) and impaired, older adults (OA-I, n=4). **B**. Diffusion metrics along the reconstructed tracts were compared between older adult (OA) and impaired, older adults (OA-I). Metrics include: fractional anisotropy (left; *, p=0.01), mean diffusivity (middle; *, p=0.05), and axial diffusivity (right; *, p=0.05) between OA and OA-I participants. Older adults = **Grey**; Impaired, older adults = **Magenta**.

We found that OA-I participants had lower distribution volumes in the MS/vDB (Figure 3), and the EC (Figure 2), that correlates with poor SVOLT-D performance. Next, we asked whether there were deficits in the structural connectivity between the MS/vDB and EC in OA-I participants compared to their OA counterparts. Using diffusion weighted MRI images acquired simultaneously to the PET imaging, we reconstructed tracts between the MS/vDB (set as seed region) and the EC (ROI/End region) (Figure 4A). We quantified diffusion metrics along the rendered tract (Figure 4A, right) between the MS/vDB and the EC in each participant. We found reductions in fractional anisotropy (FA, Figure 4B-left, p=0.01), and elevations in both mean diffusivity (MD, Figure 4B-middle, p=0.05), and axial diffusivity (AD, Figure 4B-right, p=0.05) in OA-I participants compared to OA counterparts. These data are consistent with disordered, structurally impaired tracts between the MS/vDB and EC.

### The displaced object recognition (DOR) behavioral task activates the lateral entorhinal cortex.

The data presented above establishes a relationship between EC related memory performance and the integrity of the MS/vDB cholinergic projection to the EC. To gain insight into the function of acetylcholine in the EC, we turned to animal models. We focused our questions on the cholinergic circuit to the lateral entorhinal cortex (LEC) given the LECs early role in age-related vulnerabilities^12,14,16–19^.

**Figure 5:**
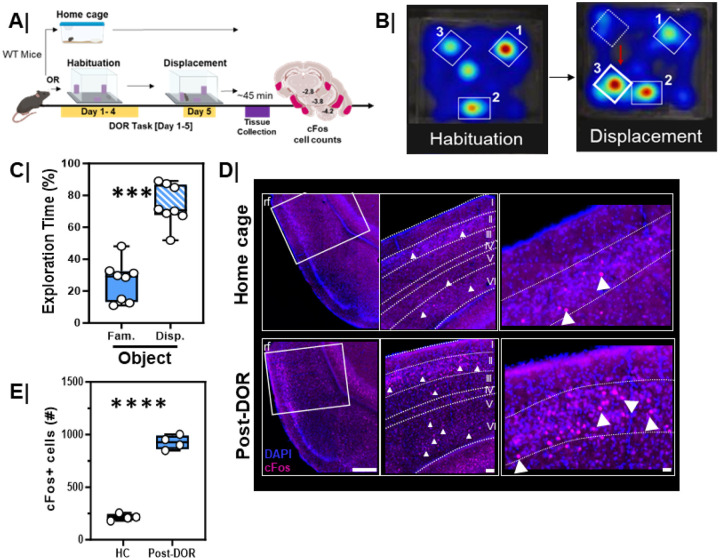
The displaced object recognition (DOR) behavioral task engages the lateral entorhinal cortex in mice **A.** (left, top) Experimental workflow. 3-month wild-type, male and female mice, were either maintained in the home cage or underwent the Displaced Object Recognition (DOR) task including habituation sessions (HAB, days 1–4) and then a displacement session (day 5). Brain tissue was harvested 45 min after the behavior session and processed for cFos immunoreactivity. **B.** Representative heat maps showing arena exploration during the habituation and displacement sessions. Red arrow denotes direction of displacement. Hot colors on the heatmap represent more time spent exploring while cool colors represent less time spent exploring. **C.** Quantification of percent object exploration in male and female mice between the two identically shaped familiar objects after one of the objects was displaced (n=8/group; Fam vs. Disp, ***, p=0.0002). **D.** (left) Low-magnification (scale 200um), (middle) mid-magnification (scale 50um), and high-magnification (scale 20 um) inset of cFos immunoreactivity in (top) home cage (HC) and (bottom) following displacement session of the DOR task. **E.** Quantification of total cFos activation in the LEC following the displacement (n=4) session of the DOR task compared to home cage (n=4) (****, p=0.0001).

In rodents, the displaced object recognition (DOR) task closely mirrors the object location task administered in human studies. We first evaluated the potential of the DOR task to engage the mouse LEC. Mice were habituated to an environment with objects in set locations over four consecutive days (Figure 5A, Habituation). On the test day, Day 5, one object was displaced to a new location within the arena (Figure 5A, Displacement). Typically, mice explore the newly displaced object more than they would the non-displaced objects, as illustrated in the sample heatmaps of Habituation vs Displacement (Figure 5B) where object 3 was moved to a new location. Indeed, during the displacement session, three-month-old WT animals spent more time exploring the displaced object compared to the familiar object (Figure 5C, p=0.0002). Total exploration did not differ between habituation and displacement sessions. Note that 3-month male and female WT mice displayed identical behaviors in this assay (**Figure S6B**, Male Fam. vs. Disp, p=0.002; Female Fam. Vs. Disp, p=0.004) so in subsequent analyses we collapsed sex as a biological variable and included both male and female mice in all experiments.

To test for activation of neurons in the LEC we assessed immunoreactivity for the expression of the immediate early gene product cFos 45 mins following the DOR task (Figure 5D/S3A). Significantly more LEC neurons expressed cFos following the DOR session when compared to mice maintained in their home cages (HC; Figure 5D/E, p=0.0001). We also evaluated cFos expression in mice following habituation for five days (HAB), following a single session of object exploration (Novel Obj.), and following exploration of an empty arena for five days (OF). Consistent with the role of the EC in object encoding^19,23,24^ we found elevated cFos immunoreactivity in the habituation and novel object groups compared to the open field and home cage control conditions (**Figure S3B**, HC/OF vs. Novel Obj/HAB, p<0.01). DOR also significantly elevated cFos in the LEC compared to novel object or habituation conditions (**Figure S3B**, Novel Obj/HAB vs. DOR, p<0.005) and open field or home cage conditions (**Figure S3B**, HC/OF vs. DOR, p<0.0001).

### 12-month-old mice have impaired DOR performance and impaired activation of the lateral entorhinal cortex.

**Figure 6:**
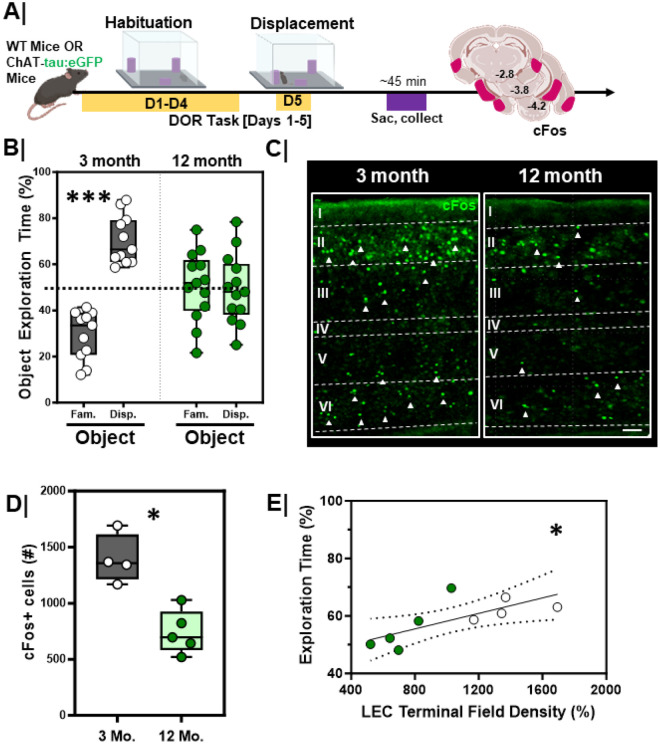
LEC activation and DOR performance is lower in aging wild-type mice **A.** Experimental workflow. WT animals were assessed in the displaced object recognition (DOR) task followed by tissue harvesting and cFos immunostaining; **B.** Quantification of percent time spent exploring familiar vs. displaced object during the displacement session in 3-month (n=11; ***, p=0.001) and 12- month animals (n=12; p=0.69). 3-month = **Grey box**; 12-month = **Green box. C.** Representative high magnification images of LEC cFos activation during the displacement session in 3-month (left) vs. 12-month (right) animals; scale 50um**D.** Quantification of total cFosactivation in the EC during the displacement test in 3-month (n=4) vs 12-month (n=5) animals (*, p=0.02). 3-month = **Grey box**; 12-month = **Green box. E.** Correlation plot with linear regression comparing the relationship between EC cFosactivation (cFos+) and DOR performance in 3-month vs 12-month animals (*, rs=0.52, p=0.03, linear regression is significantly non-zero); 3-month animals = white dots. 12-month animals = **Green dots**.

The DOR task provides a quantifiable measure of behavioral performance and engagement of the LEC^23,24^. To answer questions about the relationship between cholinergic system integrity and LEC function with age, we evaluated 12-month-old mice in the DOR task and quantified their performance as compared to 3-month-old mice (Figure 6A). Object exploration was equivalent between young and older mice during the habituation session (**Figure S4A/B**, 3 mo., p =0.85; 12 mo., p = 0.29). During the displacement session, 3-month mice spent more time exploring the displaced object (Figure 6B, Grey bars, p = 0.001), whereas 12-month mice spent about equal time in the exploration of both the displaced and familiar objects (Figure 6B, Green bars, p = 0.69). No differences were seen across sex within either the 3-month (**Figure S6B**, Male, n=10, Fam vs. Disp: **, p=0.002; Female, n=9, Fam vs. Disp: **, p=0.004; Male vs. Female: familiar object, p=0.3; displaced object, p=0.30) or 12-month-old (**Figure S6E**, Male, n=6, p=0.81; Female, n=7, p=0.69; Male vs. Female: familiar object, p=0.99; displaced object, p=0.99) mice.

To test whether altered performance was related to impaired activation of the LEC, we assessed cFos immunoreactivity following the DOR task. Mice were sacrificed 45 min following the test session (Figure 6A). 12-month animals had fewer activated neurons in the LEC following DOR than 3-month animals (Figure 6C/D, p = 0.02). No differences were seen across sex within either the 3-month (**Figure S6C**, Male, n=4 vs Female, n=4, p=0.89) or 12-month-old (**Figure S6F**, Male, n=2 vs Female, n=3, p=0.2) mice. Overall, performance on the DOR task across groups directly correlated with cFos activation in the LEC (Figure 6E, r_S_=0.52, p=0.03).

### MS/vDB cholinergic neurons project to the lateral entorhinal cortex and are activated by the DOR behavioral task.

**Figure 7:**
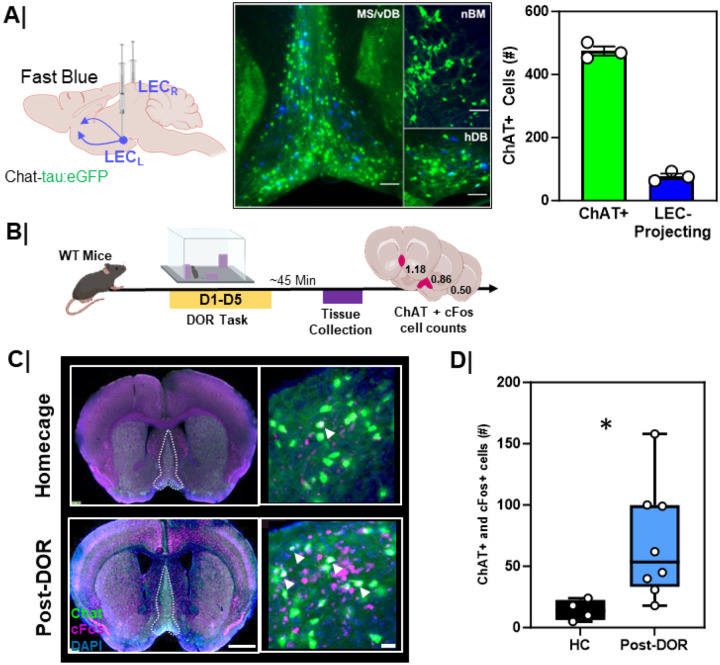
MS/vDBcholinergic neurons that project to LEC are activated by DOR **A.** (left) Schematic showing experimental workflow. Chat-tau:eGFP mice were injected with a retrograde tracer (Fast Blue) into EC followed by tissue harvesting; (middle) Representative low magnification image of LEC-projecting MS/vDBcholinergic neurons; note the predominance of blue LEC backlabeling in the medial septum/vertical diagonal band (MS/vDB) as opposed to the nucleus basalis (nBM) or horizontal diagonal band (hDB). (right) Quantification of LEC projecting cholinergic neurons in MS/vDB region of the basal forebrain as compared to total cholinergic neurons (n=3). **B.** Experimental workflow. WT mice underwent displaced object recognition (DOR) task followed by tissue harvesting and cFosimmunostaining; **C.** (left) Low magnification (scale bar = 1mm) and (right) high magnification (scale bar = 50um) inset of MS/vDB cFosactivation during the (top) home cage & (bottom) following the displacement session; White outline in low magnification images denote the MS/vDBregion. White arrows denote cFos+ and ChAT+ (activated cholinergic) neurons within the MS/vDB. **D.**Quantification of total cFosactivation in the MS/vDBfollowing the displacement (n=8) session of the DOR task compared to home cage only (n=4) (*, p=0.01).

We next investigated the origin of cholinergic input to the LEC. To identify the LEC-projecting subpopulation of cholinergic neurons, we injected with a retrograde tracer, Fast Blue, into the LEC of 3-month-old ChAT-tau:eGFP mice^31^ (Figure 7A, left). In this line, all cholinergic neurons and processes are labeled with a green fluorescent protein (GFP). We found back-labeled cholinergic neurons (Fast Blue+ and ChAT+) primarily in the MS and vDB, with the remainder in the hDB. No back-labeled cells were found in the nBM. Back-labeled cells represented about 10% of MS/vDB cholinergic neurons (Figure 7A, right). To determine whether MS/vDB cholinergic neurons were activated during the DOR task, we evaluated cFos immunoreactivity in the MS/vDB of 3-month-old wild-type mice after DOR behavioral testing (Figure 7B). Mice were sacrificed 45 min after behavior and were compared to animals that never left their home cage (Figure 7C). We found the total number of cFos+ cholinergic neurons in the MS/vDB following the displacement test (Figure 7C/D) was significantly greater compared to home cage controls (p=0.01).

### Entorhinal Cortex Terminal Field Density is Lower in 12-month mice.

**Figure 8:**
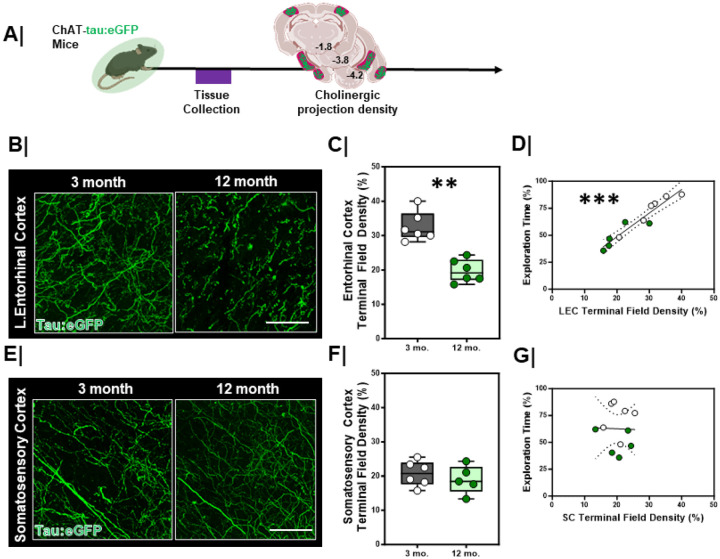
Lateral entorhinal cortex cholinergic terminal density is lower in aging mice **A.** Experimental work ow. ChAT-tau:eGFP mice were evaluated for cholinergic terminal eld integrity. **B/E.** High magnification representative confocal images of **A.** entorhinal cortex (EC) and **D.** somatosensory cortex (SC, bottom) in 3-month (left) vs. 12-month animals (right). Scale bar = 50um. **C/F.** Quantification of cholinergic terminal field density in **B.** entorhinal cortex (top, 3 mo. n=6 vs. 12 mo. n=5; **, p=0.004) and **E.** somatosensory cortex (bottom, 3 mo. n=6 vs. 12 mo. n=5; p=0.54) between 3-month and 12-month animals. 3-month = **Grey box**; 12 mo. = **Green box**. **D/G**.Correlation plots with linear regression line comparing the relationship between cholinergic terminal field density and DOR performance in **C.** the EC (left; ***, rs=0.90, p<0.001, linear regression significantly non-zero; confidence intervals denoted with dashed black lines) and **F.** in the SC (right; rs=0.0007, p=0.94, linear regression not significant; confidence intervals denoted with dashed black lines). 3-month = White dots; 12-month = **Green dots**.

Given the deficits in EC-related DOR performance, next we asked whether there were changes in the synaptic integrity of cholinergic inputs in the LEC of older mice. We used Chat-tau:eGFP mice (Figure 8A/8B) and evaluated cholinergic terminal field density in the LEC. We found that 12-month mice had significantly lower cholinergic terminal field density in the LEC than 3-month mice (Figure 8B/8C, p=0.004). In contrast, cholinergic terminal density of 12-month mice in other cortical areas such as the somatosensory cortex did not significantly differ from 3-month mice (Figure 8E/8F, p= 0.54). We did not find differences in cholinergic terminal density of the EC in the developmental period between 1.5-months and 3-months of age (**Figure S5A/S5C**, p = 0.54). No differences were found across sex within either the 3-month (**Figure S6D**, Male, n=3 vs Female, n=3, p=0.1) or 12-month-old (**Figure S6G**, Male, n=3 vs Female, n=3, p>0.99) mice. The ChAT-tau:eGFP offers a complimentary set of information to VAChT density, so we additionally compared VAChT immunoreactivity in the LEC between 12-month and 3-month animals (**Figure S5B/S5D**), offering a direct comparison to [^18^F]VAT distribution volumes in humans. We found that VAChT density was significantly lower in 12-month mice as compared to 3-month mice (**Figure S5B/S5D**, p = 0.008). These results parallel our findings with the tau:eGFP experiments and our observations using [^18^F]VAT PET in naturally aged OA-I vs. OA participants.

We also compared the relationship between cholinergic system integrity and DOR performance. We found that greater cholinergic input in the EC correlated with better performance on the DOR task (Figure 8D, r_S_ = 0.90, p<0.001). No correlation was found between somatosensory cortex cholinergic terminal field density and DOR performance (Figure 8G, r_S_ = 0.0007, p=0.94).

### MS/vDB cholinergic neurons are functionally impaired in 12-month mice.

**Figure 9:**
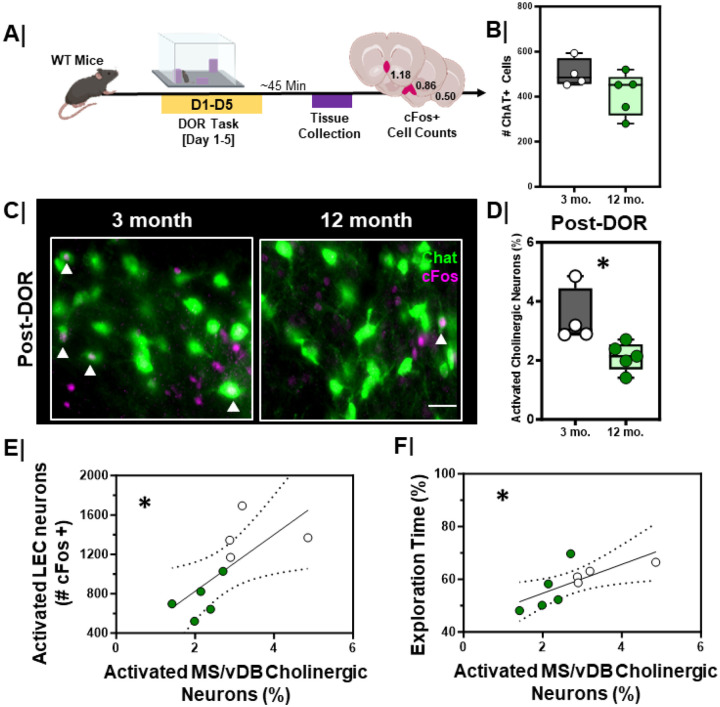
Activation of MS/vDBcholinergic neurons is lower in aging wild type mice and correlates with decreased LEC activation and poorer DOR performance **A.** Experimental work ow. WT animals were put through the displaced object recognition (DOR) task followed by tissue harvesting and cFos immunostaining; **B.** Quantification of number of ChAT+ neurons in MS/vDB of 3-month (n=4) vs. 12-month (n=5) animals (p = 0.19). 3-month = **Grey box**; 12-month = **Green box. C.** Representative high magnification images of MS/vDB cFosactivation following the displacement session of the DOR task in 3-month (left) vs. 12-month (right) animals; scale bar = 50um. White arrows denote cFos+ and ChAT+ (activated cholinergic) neurons. **D.** Quantification of % activated cholinergic neurons in the MS/DB of 3-month (n=4) vs 12-month (n=5) animals (*, p=0.02) following the displacement session of the DOR task. 3-month = **Grey box**; 12-month = **Green box. E.** Correlation plot with linear regression line comparing the relationship between percent activated cholinergic neurons (ChAT+ and cFos+) in the MS/vDB and LEC cFosactivation (cFos+) following the displacement test in 3-month (n=4) and 12-month (n=5) animals (*, rs=0.51, p=0.03, linear regression is significantly non-zero). 3-month = White dots; 12-month = **Green dots. F.** Correlation plot with linear regression comparing the relationship between percent activated cholinergic neurons (ChAT+ and cFos+) in the MS/vDBfollowing DOR displacement session and DOR performance in 3-month (n=4) vs 12-month (n=5) animals (*, rs=0.53, p=0.03, linear regression is significantly non-zero); 3-month = White dots; 12-month = **Green dots.**

By 12-months of age, in otherwise healthy mice, we observe specific deficits including lower DOR performance, lower cholinergic input to the EC, and impaired engagement of the EC following the DOR task. To test whether these changes correspond with functional changes to cholinergic neurons, we evaluated cFos expression in MS/vDB cholinergic neurons after the DOR task (Figure 9A). Although there was no significant difference in the total number of ChAT+ neurons in the MS/vDB in 3-month vs. 12-month mice (Figure 9B, p = 0.19), 12-month animals had fewer activated cholinergic neurons following DOR than 3-month animals (Figure 9C/D, p = 0.02).

We compared the relationship between cFos activation of the MS/vDB and cFos activation of the LEC following DOR (Figure 9E). We found a significant positive correlation between MS/vDB cholinergic neuron activation and LEC activation, where more cFos+ neurons in the MS/vDB correlated with more cFos+ neurons in the LEC (Figure 9E, r_S_ = 0.51, p=0.03). We also examined the relationship between cFos activation of the MS/vDB and DOR performance (Figure 9F) and found a significant positive correlation between cFos+ cholinergic neurons in the MS/vDB and performance on the DOR task (Figure 9F, r_S_ = 0.53, p=0.03).

### Aß^+^Tau^+^ mice have accelerated pathology in lateral entorhinal cortex.

**Figure 10:**
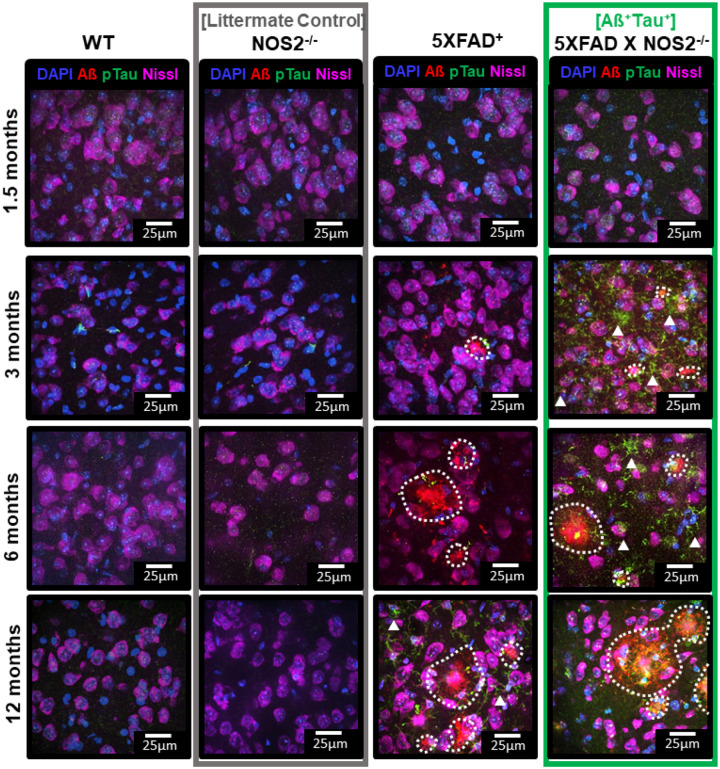
5XFAD X NOS2−/− mice have Aßand PHF-Tau accumulation in lateral entorhinal cortex by 3 months of age Representative images of Aß (red) and pTau (green) immunostaining in WT, NOS2−/−, 5XFAD+, and NOS2−/− X 5XFAD+ mice across (first row) 1.5-month, (second row) 3-month, (third row) 6-month mice from lateral entorhinal cortex with DAPI (nuclear stain, blue) and Nissl (neuronal stain, magenta) for reference. At 3 months of age and onwards both Aß and phospho-tau pathology are present in NOS2−/− X 5XFAD+ animals. Approximate outlines of Aß plaques shown by white dotted line; phosphoTau accumulation is denoted with white arrows. NOS2−/− X 5XFAD+ are herein referred to as “**Aß+Tau**+” animals. NOS2−/− mice are herein referred to as “**Controls**.” **Green vertical box** denotes images from **Aß+Tau**+ animals. **Gray vertical box** denotes images from littermate controls.

We found that deficits in the cholinergic circuit to the entorhinal cortex in natural aging of both humans and mice that correlated with impaired EC-function. Upon further examination in aged mice, we additionally found a relationship between the activation of MS/vDB cholinergic neurons, LEC neurons, and EC-function, suggesting a key role of cholinergic neurons to normal EC activation and function. Next, to answer questions about the relationship between cholinergic system integrity and LEC function in AD progression, we utilized a mouse model where deletion of NOS2 in Aß overexpressing mice (5XFAD) resulted in generation of hyperphosphorylated mouse tau (5XFAD X NOS2^−/−^, Figure 10). It was previously found that deletion of NOS2 in APP-overexpressing mice (APPSwDI, 3xFAD) led to elevated accumulation of amyloid beta plaques and spontaneous generation of hyperphosphorylated mouse tau (PHF-tau)^32–34^. We compared Aß and PHF-tau accumulation in the LEC in a 5XFAD mouse line crossed to NOS2^−/−^ mice with genetic controls (C57, NOS2^−/−^, and 5XFAD) at 1.5, 3, 6, and 12 months of age (Figure 10). Aß plaques accumulated in 5XFAD+ and 5XFAD X NOS2^−/−^ animals (Figure 10, Aß red plaques in third and fourth columns). No Aß accumulation was seen in WT or NOS2^−/−^ mice (Figure 10, first and second columns). Hyperphosphorylated-tau was only detected in 5XFAD X NOS2^−/−^, appearing by 3-months of age (Figure 10, pTau, green aggregates in fourth column vs. rest). For subsequent experiments, 3-month 5XFAD X NOS2^−/−^ (Aß^+^Tau^+^), were compared to NOS2^−/−^ littermate controls (e.g., Figure 10, littermate controls – second column vs. Aß^+^Tau^+^ mice – fourth column).

### Aß^+^Tau^+^ mice have impaired DOR performance and impaired activation of the lateral entorhinal cortex.

**Figure 11:**
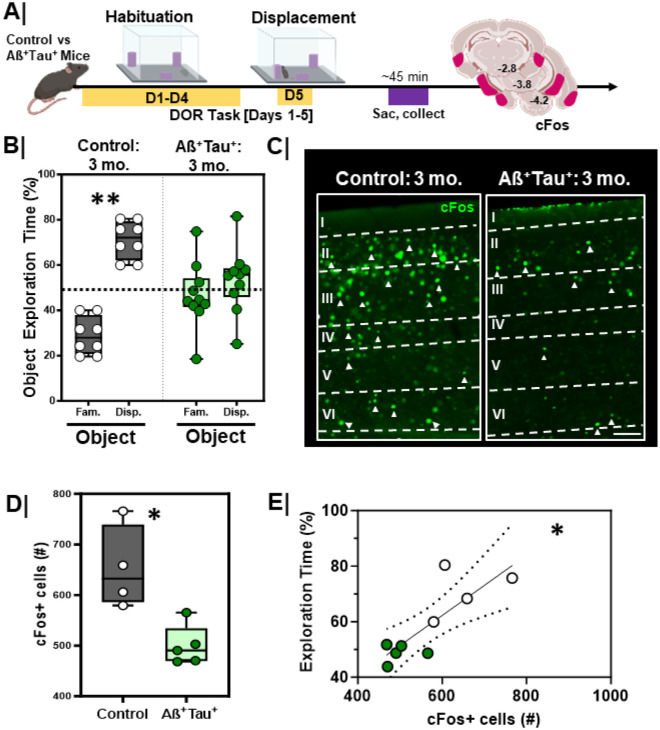
LEC activation and DOR performance is lower in Aß+Tau+ mice **A.** Experimental workflow. Aß+Tau+ animals were assessed in the displaced object recognition (DOR) task followed by tissue harvesting and cFos immunostaining; **B.** Quantification of percent time spent exploring familiar vs. displaced object during the displacement session between control (n=8; **, p=0.002) and Aß+Tau+ animals (n=10; p=0.22). Controls = **Grey box**; Aß+Tau+ = **Green box**. **C.** Representative high magnification images of LEC cFos activation during the displacement session in control (left) vs. Aß+Tau+ (right) animals; scale 50um **D.** Quantification of total cFos activation in the EC during the displacement test in control (n=4) vs Aß+Tau+ (n=5) animals (*, p=0.02). Controls = **Grey box**; Aß+Tau+ = **Green box. E.** Correlation plot with linear regression comparing the relationship between EC cFosactivation (cFos+) and DOR performance in control vs Aß+Tau+ animals (*, rs=0.73, p=0.03, linear regression is significantly non-zero); Control animals = white dots. Aß+Tau+ animals = **Green dots**.

We next quantified performance of Aß^+^Tau^+^ mice and littermate controls in the DOR task (Figure 11). As expected, exploration of objects was equivalent between groups during the habituation session (Figure S4A/C; Control, p = 0.69; Aß^+^Tau^+^, p>0.99). During the displacement session, littermate controls spent more time exploring the displaced object (Figure 11B; Grey bars, p=0.002), whereas Aß^+^Tau^+^ animals spent about equal time exploring both the displaced and familiar objects (Figure 11B; Green bars, p = 0.22).

To test whether this altered performance was related to impaired activation of the LEC, we assessed cFos immunoreactivity following the DOR task. As previously described, mice were sacrificed 45 min following the DOR test session (Figure 11A). Aß^+^Tau^+^ animals had fewer activated neurons in the LEC following DOR than littermate controls (Figure 11C/D, p=0.02), with overall performance in DOR directly correlating with cFos+ cells in the LEC (Figure 11E, r_S_=0.73, p=0.03).

### Entorhinal Cortex Terminal Field Density is Lower in Aß^+^Tau^+^ mice.

**Figure 12:**
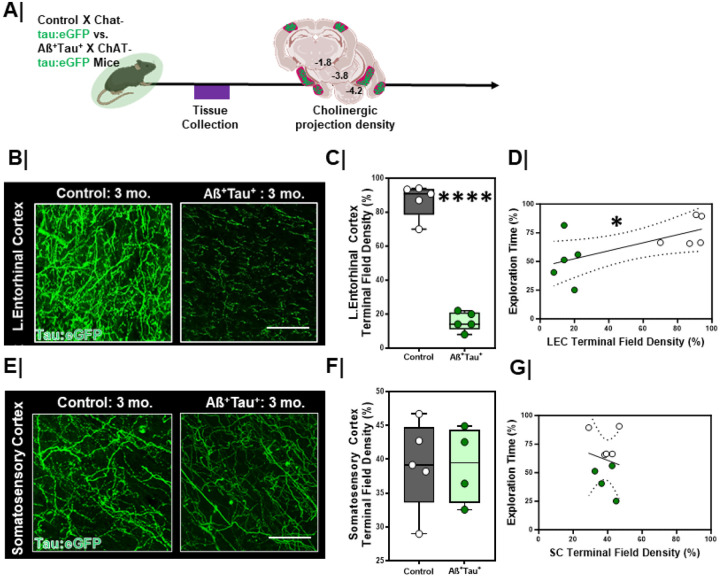
Lateral entorhinal cortex cholinergic terminal density is lower in Aß+Tau+ mice **A.** Experimental workflow. Aß+Tau+ animals were crossed to ChAT-tau:eGFP mice for evaluation of cholinergic terminal field integrity. **B/E.** High magnification representative confocal images of **B.** entorhinal cortex (EC) and **E.** somatosensory cortex (SC, bottom) in control (left) vs. Aß+Tau+ animals (right). Scale bar = 50um. **C/F.** Quantification of cholinergic terminal field density in **C.** entorhinal cortex (top, n=5/group; ****, p<0.0001) and **F.** somatosensory cortex (bottom, control n=5 + Aß+Tau+ n=4; p=0.90) between control and Aß+Tau+ animals. Controls = **Grey box;** Aß+Tau+ = **Green box**. **D/G.** Correlation plots with linear regression line comparing the relationship between cholinergic terminal eld density and DOR performance in the **D.** EC (left; *, rs=0.64, p=0.05, linear regression significantly non-zero; confidence intervals denoted with dashed black lines) and **G.** SC (right; rs=0.15, p=0.69, linear regression not significant; confidence intervals denoted with dashed black lines). Controls = White dots; Aß+Tau+ = **Green dots**.

Given the deficits in EC-related DOR performance, next we asked whether there were changes in the synaptic integrity of cholinergic inputs in the LEC in Aß^+^Tau^+^ mice. We crossed Aß^+^Tau^+^ to Chat-tau:eGFP mice (Figure 12A/12B) and evaluated cholinergic terminal field density in the LEC. We found that Aß^+^Tau^+^ animals had significantly lower cholinergic terminal field density in the LEC at 3-months compared to littermate controls (Figure 12B/12C, p<0.0001). In contrast, cholinergic terminal field density in other cortical areas such as the somatosensory cortex did not significantly differ between groups (Figure 12E/12F, p= 0.90). We found that cholinergic terminal field density in Aß^+^Tau^+^ mice did not differ from littermate controls at 1.5 months (**Figure S6A/S6C, left column**, p=0.55), but did differ by 3 months (Figure 12B/12C **and Figure S6A/S6C, right column**, p<0.0001). These differences persisted through 12-months (**Figure S6A/S6C, right column**, p=0.008) We additionally compared VAChT immunoreactivity in the LEC between Aß^+^Tau^+^ and littermate controls (**Figure S6B/S6D**). We found that VAChT density was significantly lower in 3-month-old Aß^+^Tau^+^ mice as compared to littermate controls (**Figure S6B/S6D** p=0.03). Next, we compared the relationship between cholinergic system integrity and DOR performance. We found that greater cholinergic input in the EC correlated with better performance on the DOR task (Figure 12D, r_S_ = 0.64, p=0.05), whereas no correlation was found between somatosensory cortex cholinergic terminal field density and DOR performance (Figure 12G, r_S_ = 0.15, p=0.69). These results in young, genetically susceptible animals parallel our findings in aging wild type mice and our observations using [^18^F]VAT PET in OA-I vs. OA participants highlighting the loss of cholinergic integrity in the LEC as a conserved early indicator of cognitive impairment.

### Baseline Entorhinal Cortex Activity is Elevated in Aß+Tau+ mice.

Aß^+^Tau^+^ mice displayed poor performance on the DOR task and blunted activation (as measured by cFos immunoreactivity) in the LEC following DOR. We also found that Aß^+^Tau^+^ mice have lower cholinergic input to the LEC. To test whether these changes were accompanied by changes to baseline firing rates in the LEC, we evaluated the activity in LEC in Aß^+^Tau^+^ mice compared to littermate controls using in vivo recording in anesthetized mice (**Figure S6E**). Electrodes were placed in the LEC in control and Aß^+^Tau^+^ mice and single unit baseline activity was recorded. We found LEC recordings from Aß^+^Tau^+^ mice displayed elevated firing rate, with a highly disorganized firing pattern, compared to control animals (**Figure S6F/G**, p=0.012).

### MS/vDB cholinergic neurons are functionally impaired in Aß^+^Tau^+^ mice.

**Figure 13:**
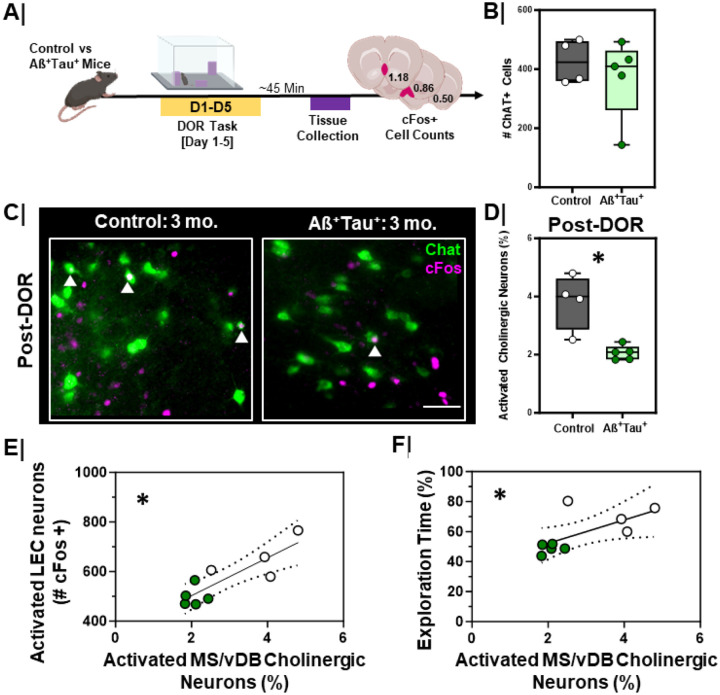
Activation of MS/vDB cholinergic neurons is lower in Aß+Tau+ animals and correlates with lower LEC activation and poorer DOR performance **A.** Experimental work ow. Aß+Tau+ animals were put through the displaced object recognition (DOR) task followed by tissue harvesting and cFos immunostaining; **B.** Quantification of number of ChAT+ neurons in MS/vDB of control (n=4) vs. Aß+Tau+ (n=5) animals (p=0.90). Controls = **Grey box**; Aß+Tau+ = **Green box. C.** Representative high magnification images of MS/DB cFos activation following the displacement session of the DOR task in control (left) vs. Aß+Tau+ (right) animals; scale bar = 50um. White arrows denote cFos+ and ChAT+ (activated cholinergic) neurons. **D.** Quantification of % activated cholinergic neurons in the MS/vDB of control (n=4) vs Aß+Tau+ (n=5) animals (*, p=0.02) following the displacement session of the DOR task. Controls = **Grey box**; Aß+Tau+ = **Green box**. **E.** Correlation plot with linear regression line comparing the relationship between percent activated cholinergic neurons (ChAT+ and cFos+) in the MS/vDB and LEC cFosactivation (cFos+) following the displacement test in control (n=5) and Aß+Tau+ (n=5) animals (*, rs=0.77, p=0.02, linear regression significantly non-zero). Controls = White dots; Aß+Tau+ = **Green dots. F.** Correlation plot with linear regression comparing the relationship between the percent activated cholinergic neurons (ChAT+ and cFos+) in the MS/vDB following DOR displacement session and DOR performance in control (n=4) vs Aß+Tau+ (n=5) animals (*, rs=0.80, p=0.01, linear regression is significantly non-zero); Controls = White dots; Aß+Tau+ = **Green dots**.

Aß^+^Tau^+^ mice display impaired DOR performance, lower cholinergic input in the EC, impaired engagement of the EC following DOR, and altered patterns of EC firing as measured by single unit recordings. To test whether these changes corresponded with functional changes to MS/vDB cholinergic neurons following DOR performance, we evaluated cFos activation of MS/vDB cholinergic neurons in the Aß^+^Tau^+^ mice after DOR performance (Figure 13A). As previously described, mice were sacrificed 45 min after the test session (Figure 13A). Although we found no differences in the total number of MS/vDB ChAT+ neurons in control vs. Aß^+^Tau^+^ mice (Figure 13B, p=0.90), Aß^+^Tau^+^ mice had fewer activated (cFos+) cholinergic neurons following DOR than littermate controls (Figure 13C/D, p=0.02).

We next evaluated the relationship between cFos activation of the MS/vDB, cFos activation of the LEC, and DOR performance (Figure 13E/F). We found a significant positive correlation between the percent activated MS/vDB cholinergic neurons and the activation of the LEC (Figure 13E, r_S_ = 0.77, p=0.02). We also found a significant positive correlation between cFos+ cholinergic neurons in the MS/vDB and performance on the displacement day of the DOR task (Figure 13F, r_S_ = 0.80, p=0.01).

### Lateral entorhinal cortex projecting cholinergic neurons are necessary for proper DOR performance.

**Figure 14:**
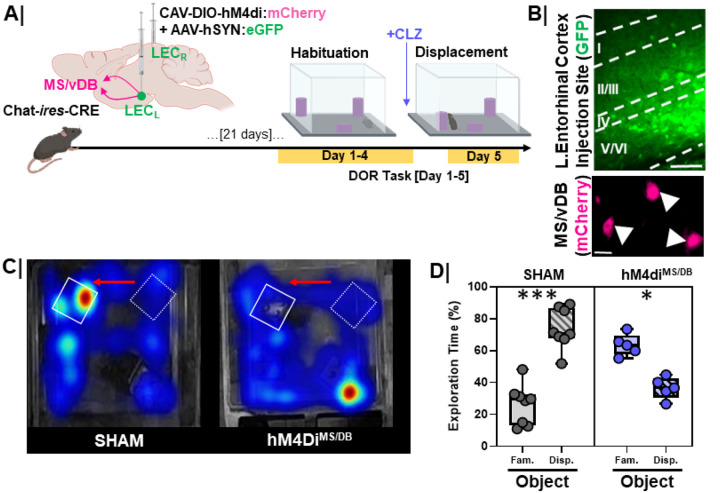
Silencing lateral entorhinal cortex projecting cholinergic neurons in young WT mice impairs DOR performance **A.** Experimental work ow. Young, 3-month-old, chat-creanimals were injected with a retrograde inhibitory DREADD construct (CAV-DIO-hM4di:**mCherry**) and a GFP control virus (AAV-hSYN:**eGFP**)to mark the injection site in LEC for cell-type specific and projection specific, manipulation of cholinergic signaling (to the EC) during the displaced object recognition task (DOR). Clozapine (0.1 mg/kg) was administered via i.p.injection (to activate the DREADD) 10 minutes prior to the displacement session (day 5); **B.** (Top) Representative image of LEC injection site, scale bar = 50um. (Bottom) Representative image of hM4Di-expressing neurons in the MS/vDB, demonstrating successful, Cre-specific back-labeling of EC-projecting cholinergic neurons, scale bar = 25um; **C.** Representative heatmap showing arena exploration in control sham (left) vs. hM4diMS/vDB inhibition group; Red arrows denote direction of displacement. Hot colors on the heatmap represent more time spent exploring while cool colors represent less time spent exploring. **D.** Quantification of percent object exploration between the objects in control (n=7; ***, p=0.0002) vs. hM4diMS/vDB (n=5; *, p=0.008) mice. Data indicate the preference for the displaced object is blocked by inhibition of cholinergic projection neurons residing in the MS/vDB. Controls = **Grey box**; hM4diMS/DB = **Purple box**.

Activation of cholinergic neurons correlated with both EC activation during DOR, and normal DOR performance. To determine whether cholinergic input to LEC is required for normal DOR performance, we injected the LEC of Chat-IRES-Cre-Δneo mice with CAV_2_-DIO-hM4Di.mCherry and AAV_9_-hSyn-eGFP (DREADDi animals) or AAV_9_-hSyn-eGFP alone (control animals) (Figure 14A). DREADDi and control animals were administered clozapine (CLZ, i.p.) at concentrations sufficient for selective activation of the DREADDi 10 minutes prior to the object displacement test session (Figure 14A). Tissue was processed for relocalization of the injection site and verification of mCherry+ cells in the MS/vDB (Figure 14B). Clozapine injection had no significant effect on control animal behavior; animals spent more time exploring the displaced object than the familiar object (Figure 14C/D **grey bars**, p=0.0002). Inhibiting MS/vDB cholinergic neurons resulted in less time exploring the displaced object compared to the familiar object (Figure 14C/D **purple bars**, p=0.008). Thus, activity of entorhinal cortex-projecting cholinergic neurons was required for proper DOR performance.

## III. Discussion

In this detailed PET/MRI study of 14 aging humans and parallel anatomical and functional experiments on over 100 healthy aging and genetically modified mice, we find that cholinergic input from the MS/vDB to the EC begins to deteriorate at early stages of cognitive impairment in a mechanism that is shared across both normal and pathological aging. In both species, the decrease in cholinergic terminal field integrity in the EC correlates with impaired performance on EC related object location memory tasks. Furthermore, selective manipulations of the cholinergic system in young mice impairs object location memory, mimicking age-related disturbances to EC function. These data are consistent with a primary role of acetylcholine in the cognitive deficits associated with early EC dysfunction in age-related cognitive decline.

### Loss of Cholinergic Input in the [Lateral] Entorhinal Cortex is an Early Feature of Cognitive Decline in natural and pathological aging

Prior post-mortem studies report loss of cholinergic markers and neurons in individuals with Alzheimer’s disease^5–7^. Using recent advances in in vivo imaging methodology^25–27^, we evaluated cholinergic synaptic integrity in the human EC, a region that is uniquely susceptible to aging and neurodegenerative disease. We find that loss of cholinergic input to the EC is, in fact, an early occurrence in the progression of cognitive decline in in otherwise healthy, aging humans and mice, revealing a specific vulnerability in the circuit between the MS/vDB (CH1/2) of the basal forebrain and the EC. We find that the deterioration of cholinergic input to the EC is also apparent, and in fact accelerated in animals that are genetically susceptible to early Alzheimer’s disease.

It is well established that EC function is required for object location memory in primates and in rodents^17,19,24,35–37^. Likewise, projection patterns of the basal forebrain cholinergic system are conserved across species^3,38,39^. Here we find that in addition to the anatomical connectivity and functional role of the EC, the function of the EC-projecting cholinergic circuit, and the age-related vulnerability of this circuit is also conserved in humans and mice. As such, cross-species investigation of the underlying mechanisms of age-related changes in cognition are of clinical relevance.

In mouse studies, we evaluated the vulnerability of the EC-projecting cholinergic circuit in both healthy aging animals and animals with genetic susceptibility to accelerated amyloid plaque and hyperphosphorylated tau pathology. Remarkably, we found that even with this accelerated pathology, *the same* cholinergic circuit is affected in a similar manner, underscoring specific vulnerability of this circuit in normal and pathological aging, albeit at different rates. It is reasonable to assume, with further evaluation at later timepoints that additional cholinergic regions and circuits could be affected in the genetically susceptible or naturally aged animals that differ in onset and trajectory. Yet, it remains striking that the inherent vulnerability in the circuit encompassing the MS/vDB and the EC is conserved across these very different mechanisms of aging with a difference in age of onset.

In human studies (wherein possible) we also evaluated apolipoprotein E (*APOE*) genotype. The ApoE protein is critical in mediating lipid homeostasis and is thought to play a role in amyloid plaque aggregation and clearance in the brain^40,41^. Genome-wide association studies suggest the ε4 allele of the APOE gene is one of the strongest risk factors for dementia and AD and earlier age of onset^40,42^. In the present study, in evaluated samples, we find a higher occurrence of ε4 heterozygotes in the OA-I group as compared to the OA group consistent with the potential for elevated risk of cognitive impairment in ε4 carriers. This population was recruited as otherwise healthy and yet without a clinical diagnosis of cognitive impairment. Despite this, we are already able to identify loss of integrity of EC cholinergic input and EC-related cognitive deficits, highlighting the potential importance of the integrity of this circuit as an early diagnostic marker.

### Functional Changes in the Basal Forebrain Projection to the Entorhinal Cortex Precede Cell Loss

We evaluated the integrity of basal forebrain cholinergic nuclei in our human cohort using an established segmentation atlas^30^. The distribution of [^18^F]VAT accurately reports VAChT expression in the brain, and can be used to measure cholinergic projections as well as cholinergic cell bodies^43^. We find that early in the progression of cognitive impairment, MS/vDB (CH1/2) cholinergic integrity (volume of distribution) is lower in OA-I participants. Though our data are cross-sectional, this likely reflects changes in VAChT levels in cholinergic soma and/or local axons. We did not nd decreased cholinergic integrity in hDB (CH3) or nBM/SI (CH4) nuclei.

Leveraging higher resolution imaging capability in mice, we were able to use ChAT immunoreactivity to count MS/vDB cholinergic neurons and found no differences between 3-month and 12-month animals or control and cognitively impaired Aß^+^Tau^+^ mice. Although the numbers of MS/vDB cholinergic neurons did not differ, the engagement of MS/vDB cholinergic neurons by object location memory was significantly lower in mice with impaired DOR performance. If conserved across species, this suggests that lower MS/vDB volume of distribution found in OA-I participants reflects decreased VAChT expression, and not a loss of cholinergic neuron number per se.

A prior seed-to-searchlight MRI analysis found that basal forebrain nuclear degeneration covaries with cortical degeneration, reflective of their projections^44^. Our data are consistent with this hypothesis that the local EC cholinergic terminal integrity and the integrity of EC-projecting cholinergic circuits are amongst the earliest affected. Our targeted diffusion imaging analysis of the structural integrity of the circuit between the MS/vDB and the EC in humans was consistent with disordered, fragmented, and structurally impaired connectivity. Affected parameters included decreases in the anisotropic diffusion and increases in mean and axial diffusivity, consistent with a loss of white matter integrity as reported in neurodegenerative conditions^45^ and advanced age^46^.

### Acetylcholine Plays a Critical role in Entorhinal Cortex Function and Performance

The EC receives a dense projection from the medial septum and diagonal band nucleus consisting of glutamatergic, GABAergic, and cholinergic input^47^. Cholinergic terminals in the LEC synapse onto both principal neurons and interneurons that express varied muscarinic and nicotinic acetylcholine receptors^48^. As a result of this variation, the net activity of acetylcholine in the LEC is complex and is likely to be coordinated in a behaviorally relevant manner. In our mouse studies, we find that there are significant correlations between the extent of MS/vDB cholinergic activation, the integrity of their projections to the EC, and the activation of EC neurons and DOR performance. Perhaps most compelling, chemogenetic inhibition of EC projecting cholinergic neurons in healthy young mice disrupts DOR performance. Our results are consistent with the hypothesis that appropriate ACh tone in the EC is important for proper EC function.

Aß^+^Tau^+^ animals display elevated baseline activity compared to littermate controls. This finding of hyperexcitability in aging circuits is congruent with a growing body of literature^14,49,50^. There are several mechanisms that could underlie this phenotype including Aß accumulation contributing to downstream synaptic dysfunction^51^, imbalance of the excitation:inhibition balance^51^ potentially due to loss of cholinergic input, and an overall decrease in GABAergic tone^49^. We propose that loss of EC ACh tone (loss of cholinergic input) results in elevation of circuit activity (increased baseline excitability), and an inability to specifically activate the EC in a behaviorally relevant manner (impaired cFos activation of MS/DB and EC), resulting in impaired object location memory performance. In support of this, silencing EC-projecting cholinergic input in normal animals is sufficient to dramatically affect object location memory performance.

### Quantifying Vesicular Acetylcholine Transporter In Vivo in Humans using [^18^ F]VAT

Reliable quantification of cholinergic nuclei and terminal fields is possible using PET tracers that target the vesicular acetylcholine transporter (VAChT). Two such probes have recently been developed: [^18^F]FEOBV^52^ and [^18^F]VAT^25^. FEOBV has been used in rodents^53,54^, nonhuman primates^54^, and healthy human volunteers^52,44,55–57^. Quantifying FEOBV is limited by slow kinetics that necessitate using either long scan times or short semi-quantitative static scans that rely on estimates of non-equilibrium tissue ratios including standardized uptake value ratios (SUVR). Tu et al generated [^18^F]VAT by modifying FEOBV’s structure. Early studies in rodents^25^ and non-human primates^26,27^ demonstrate that VAT has the faster kinetics necessary for fully quantitative measurement of VAChT throughout the brain. Based on these findings, we chose to utilize [^18^F]VAT for in vivo PET imaging of cholinergic terminal field integrity in an elderly population of humans. Using metabolite-corrected arterial plasma [^18^F]VAT concentration as input, we estimated VAChT distribution volume in key regions of interest throughout the brain. We were able to demonstrate differential terminal field loss in entorhinal cortex vs. hippocampus and somatosensory cortex in subjects who showed cognitive deficits on both the MOCA and the PennCNB SVOLT tests. Notably, these individuals were not recruited based on an existing clinical diagnosis but were part of a healthy community-based cohort. These results indicate that there are quantifiable losses in cholinergic terminal integrity in the EC in healthy individuals that correlate with reduced performance on cognitive tasks.

Previous studies evaluated cortical FEOBV SUVR in participants recruited with a diagnosis of MCI^56^ or AD^55^. These studies reported global deficits in cortical cholinergic innervation in individuals in the MCI or AD groups^56^. We evaluated a population without clinical diagnosis of cognitive impairment, albeit with subjective memory concerns. Our studies extend upon existing findings to assess the integrity of cholinergic circuitry early in cognitive impairment and probe the cholinergic mechanisms underlying impaired EC function.

It has previously been found that VAChT uptake was a better predictor of Alzheimer’s disease than either amyloid beta load ([^18^F]^−^NAV uptake) or brain glucose metabolism ([^18^F]-FDG uptake)^55^. We focused our a priori analyses in both humans and mice on the circuit between the basal forebrain and EC based on reported early issues with EC-related functions with age^17,19^. We found that loss of cholinergic input to the entorhinal cortex is an early phenomenon in cognitive decline as assessed in otherwise healthy aging humans and mice, as well as in mice with genetic susceptibility to amyloid plaque accumulation. Our results are consistent with the hypothesis that loss of EC cholinergic terminal density might underlie some of the earliest phases of age-related cognitive decline. At these early stages of EC-related cognitive impairment, loss of cholinergic terminal density in the EC might precede and be decoupled from amyloid plaque accumulation. As such, we propose that the EC-specific cholinergic deficits likely precede the more robust global cortical deficits found in previous studies. Our ongoing longitudinal studies with larger participant cohorts and improved PET resolution are investigating the importance of LEC-specific cholinergic terminal integrity as the earliest predictive factor of future cognitive impairment. We suggest that VAChT density as assessed by [^18^F]-VAT could be useful as an early predictive measure of age-related cognitive impairment.

### Conclusions

In a series of translational experiments in healthy aging humans, healthy aging mice, and in mice with genetic susceptibility to Alzheimer’ disease, we present data supporting loss of cholinergic innervation in the LEC and loss of function of LEC-projecting cholinergic neurons as an early step in the aging trajectory and intimately related to early cognitive decline. Furthermore, we reveal an important role for ACh in normal EC functional engagement and object location memory. Our data suggests EC VAChT availability may be a sensitive biomarker for early detection and potential intervention in age-related cognitive decline. If the goal of the field is to find biomarkers for early intervention, it seems we are still looking too late! At this early stage of cognitive impairment, the BFCN◊EC circuit is already affected. Using these valuable in vivo imaging tools, supported by parallel preclinical assessment, studies that evaluate the onset of the cholinergic lesion and understand the predictive capability of VAChT density in diagnosing future cognitive impairment are needed. In addition, we find that while some cholinergic circuits are dramatically affected at this point, some remain intact. This concept falls in line with a growing body of literature supporting the heterogeneity of different central cholinergic populations^1,58,59^. Understanding what confers differential resilience and vulnerability to these populations may be key in maintaining cognition long-term.

## Figures and Tables

**Table 1:** Sample Characteristics

	Older Adults	Impaired, Older Adults	P-value
Sample Metrics			
**Total Participants**	**9**	**5**	--
**Age (years)**	**64.98** ± 7.7	**69.47** ± 8.8	0.28
**Sex (% female)**	**37.50%**	**100.00%**	0.01*
*APOE* Geno typing			
**ε3/ε3**	**3 participants**	**1 participant**	
**ε3/ε4**	**1 participant**	**3 participants**	--
Cognitive Measures			
**MMSE (score out of 30)**	**28.78** ±1.1	**25.2** ± 3.7	0.09
**MoCA (score out of 30)**	**27.56** ± 1.3	**21.4** ± 4.2	0.005*
**PennCNB: SVOLT (score out of 30 trials)**	**15.44** ± 1.6	**15.2** ± 1.8	0.8
**PennCNB SVOLT-D (score out of 30 trials)**	**15.11** ± 1.2	**10.8** ± 3.4	0.01*
Imaging Metrics			
**Injected Dose [** ^**18**^ **F]VAT (mCi)**	**3.84** ± 0.8	**3.77** ± 0.6	0.14
**Free Fraction (f**_**P**_)	**0.03** ± 0.003	**0.03** ± 0.01	0.44
**FreeSurfer Cortical Thickness: Entorhinal Cortex (mm)**	**3.15** ± 0.4	**3.09** ± 0.6	0.81
**FreeSurfer Cortical Volume: Entorhinal Cortex (mm** ^**3**^ **)**	**1475.67** ± 343.2	**1260.88**± 271.6	0.13

## Data Availability

The datasets generated and analyzed in this study are available from the corresponding authors on reasonable request.

## Code Availability Statement

No custom code was generated in this study. The pipeline used to generate human imaging results has been described in the methods section.
